# Whole-transcriptome analysis links trastuzumab sensitivity of breast tumors to both HER2 dependence and immune cell infiltration

**DOI:** 10.18632/oncotarget.4405

**Published:** 2015-07-01

**Authors:** Tiziana Triulzi, Loris De Cecco, Marco Sandri, Aleix Prat, Marta Giussani, Biagio Paolini, Marialuisa L. Carcangiu, Silvana Canevari, Alberto Bottini, Andrea Balsari, Sylvie Menard, Daniele Generali, Manuela Campiglio, Serena Di Cosimo, Elda Tagliabue

**Affiliations:** ^1^ Department of Experimental Oncology and Molecular Medicine, Molecular Targeting Unit, Fondazione IRCCS Istituto Nazionale dei Tumori, Milan, Italy; ^2^ Department of Experimental Oncology and Molecular Medicine, Functional Genomics Core Facility, Fondazione IRCCS Istituto Nazionale dei Tumori, Milan, Italy; ^3^ Translational Genomics Group, Vall d’Hebron Institute of Oncology, Barcelona, Spain; ^4^ Medical Oncology Department, Hospital Clínic i Provincial, Barcelona, Spain; ^5^ Department of Pathology, Anatomic Pathology A Unit, Fondazione IRCCS Istituto Nazionale dei Tumori, Milan, Italy; ^6^ Dipartimento di Terapia Molecolare e Farmacogenomica, Istituti Ospitalieri di Cremona, Italy; ^7^ Dipartimento di Scienze Biomediche per la Salute, Università degli Studi di Milano, Italy; ^8^ Department of Oncology, Fondazione IRCCS Istituto Nazionale dei Tumori, Milan, Italy

**Keywords:** breast cancer, trastuzumab benefit, gene expression profiling, lymphocytes

## Abstract

While results thus far demonstrate the clinical benefit of trastuzumab, some patients do not respond to this therapy. To identify a molecular predictor of trastuzumab benefit, we conducted whole-transcriptome analysis of primary HER2+ breast carcinomas obtained from patients treated with trastuzumab-containing therapies and correlated the molecular portrait with treatment benefit.

The estimated association between gene expression and relapse-free survival allowed development of a trastuzumab risk model (TRAR), with *ERBB2* and *ESR1* expression as core elements, able to identify patients with high and low risk of relapse. Application of the TRAR model to 24 HER2+ core biopsies from patients treated with neo-adjuvant trastuzumab indicated that it is predictive of trastuzumab response. Examination of TRAR in available whole-transcriptome datasets indicated that this model stratifies patients according to response to trastuzumab-based neo-adjuvant treatment but not to chemotherapy alone. Pathway analysis revealed that TRAR-low tumors expressed genes of the immune response, with higher numbers of CD8-positive cells detected immunohistochemically compared to TRAR-high tumors.

The TRAR model identifies tumors that benefit from trastuzumab-based treatment as those most enriched in CD8-positive immune infiltrating cells and with high *ERBB2* and low *ESR1* mRNA levels, indicating the requirement for both features in achieving trastuzumab response.

## INTRODUCTION

Trastuzumab is the standard-of-care treatment for patients with HER2+ breast cancer (BC), reducing the risk of relapse and death in patients when administered with chemotherapy [1]. Even so, some patients do not benefit from this reagent and disease recurs [2, 3]. Thus, HER2 expression as determined by immunohistochemistry or fluorescence *in situ* hybridization is insufficient for selection of patients likely to benefit from this therapy, indicating the need to identify a biomarker(s) able to recognize such patients. Retrospective analyses from major studies of trastuzumab treatment have suggested that tumor dependence on HER2 or immune infiltrate might serve as predictive biomarkers. Two studies that used expression profiling of selected genes in archived formalin-fixed, paraffin-embedded (FFPE) tumor blocks support the significance of *ERBB2* mRNA expression in predicting trastuzumab benefit [4, 5], and evidence for the predictive value of tumor-infiltrating lymphocytes is emerging [6, 7].

To determine whether whole-transcriptome analysis of HER2+ primary BCs might improve the search for molecular features predictive of trastuzumab benefit, we conducted gene expression profiling of archived FFPE tumor blocks from HER2+ BCs. A model constructed based on genes strictly associated with relapse-free survival (RFS) identified two subgroups of HER2+ BC with distinct biological characteristics that benefit differently from trastuzumab-based therapy both in adjuvant and neo-adjuvant settings. Responsive tumors were enriched both in HER2 dependent signals and in immune cell infiltration.

## RESULTS

### Construction of a model for risk of relapse

**Figure 1:**
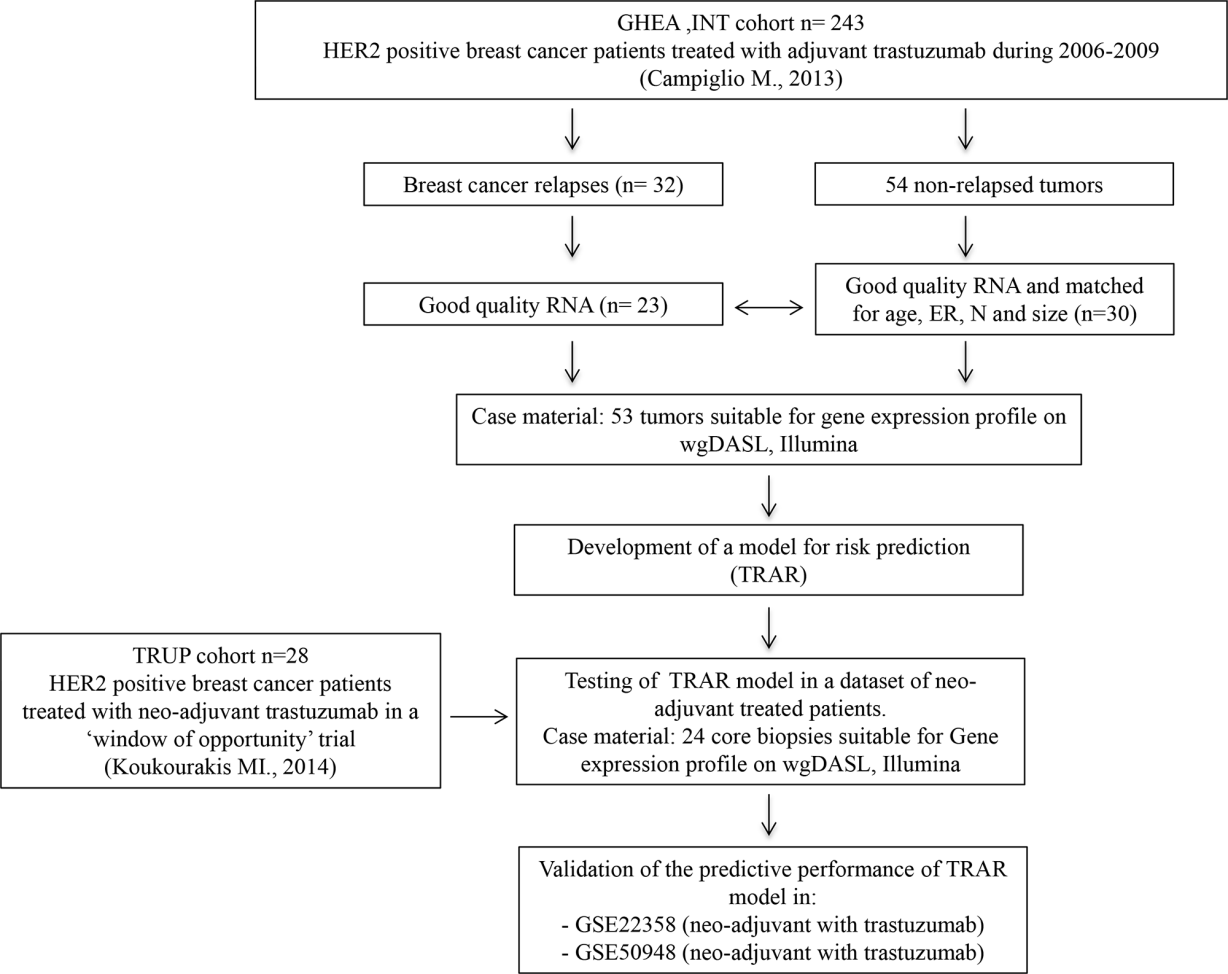
CONSORT diagram of the study GHEA, Group HErceptin in Adjuvant Therapy [17]. ER, estrogen receptor; N, lymph node status.

**Figure 2:**
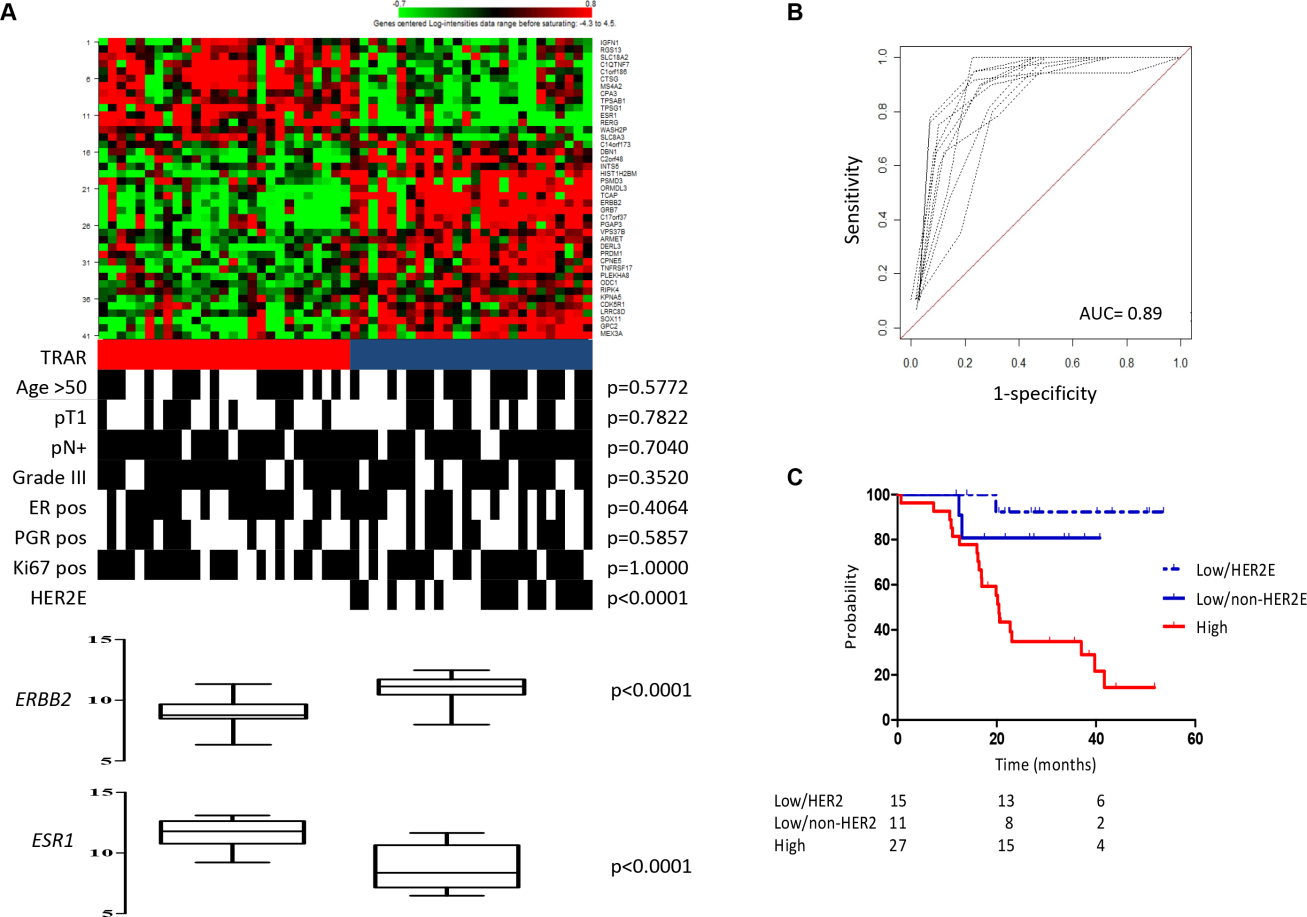
Development of 41-gene risk model **A.** Heat-map of 41-gene model expression and TRAR classification (red, high risk; blue, low risk). Clinical and pathological characteristics are shown. pN, lymph node; ER, estrogen receptor; PGR, progesterone receptor. *p*-values by Fisher’s exact test. **B.** In-sample prediction performance of the classifier. Receiver-operator characteristics (ROC) curves were based on high- and low-risk classes computed using the 41-gene model on 10-fold cross-validation. **C.** Association between TRAR-high (red) and -low (HER2E, dotted blue; non-HER2E, blue) patients with RFS.

To test whether whole-transcriptome expression profiling of HER2+ BCs can identify a biomarker indicating benefit from adjuvant trastuzumab, we analyzed the gene expression profile of 53 tumors and developed the TRAstuzumab Risk (TRAR) prediction model (Figure 1). Using a semi-supervised principal component method, we identified patients with high and low risk of relapse (Figure 2A). Based on a threshold defined by a 10-fold cross-validation method [8], samples were grouped as high (*n* = 27) or low (*n* = 26) risk of early relapse, as confirmed by survival analysis revealing an 8-fold higher risk of relapse in the high- versus low-risk group in this selected cohort (HR = 8.0, 95% CI = 3.5–18.2, *p* = 0.0001). The model had a good performance (Figure 2B) and the classification was independent of clinico-pathological characteristics (Figure 2A). Among the 41 genes of the model (listed in Table S1), 9 that persisted in the model during permutation tests to define the relative weight of each gene represented a core element of TRAR. Six of these genes were associated with HER2 (*ERBB2*, *GRB7*, *ORMDL3)* or ER (*C1orf186*, *ESR1*, *RERG*). *ERBB2*-related genes were more highly expressed in low-risk than in high-risk patients, whereas the opposite was found for *ESR1*-associated genes (Figure 2A).

Based on the apparent relevance of *ERBB2* and *ESR1* mRNA levels in discriminating patients with low or high risk of relapse, we applied to our dataset the PAM50 subtype predictor, which identifies the HER2-enriched (HER2E) subtype as the tumor group most responsive to trastuzumab [5]. Kaplan-Meier analysis confirmed that patients with HER2E tumors had the best survival outcome after adjuvant trastuzumab therapy compared to all other collective subtypes in our cohort (*p* = 0.0020, Figure S1). PAM50 classification was significantly associated with TRAR (*p* < 0.0001), with all HER2E tumors classified as TRAR-low (Figure 2A) but not all TRAR-low classified as HER2E. Kaplan-Meier analysis stratifying TRAR-low tumors into HER2E and non-HER2E indicated that both had similar recurrence probability and a significantly lower recurrence probability than TRAR-high tumors (TRAR-low/non-HER2E vs TRAR-high: *p* = 0.0312, TRAR-low/HER2E vs TRAR-high: *p* = 0.0003, Figure 2C).

To test whether the TRAR model identifies patients with intrinsic poor prognosis independent of trastuzumab treatment, we analyzed 132 HER2+ BCs treated with adjuvant chemotherapy alone from the Metabric dataset. We found only borderline statistical significance toward better prognosis for TRAR-high versus TRAR-low tumors (HR = 0.64; 95% CI: 0.38–1.08, *p* = 0.0986; Figure S2), suggesting that TRAR-low tumors have a bad prognosis if treated with chemotherapy alone and thus that the model is specific for the benefit from trastuzumab treatment. Notably, analysis of TRAR-low partitioned according to PAM50 indicated similar recurrence rates for both HER2E and non-HER2E tumors when treated with chemotherapy alone (Figure S2).

### Predictive power of the TRAR model

**Figure 3:**
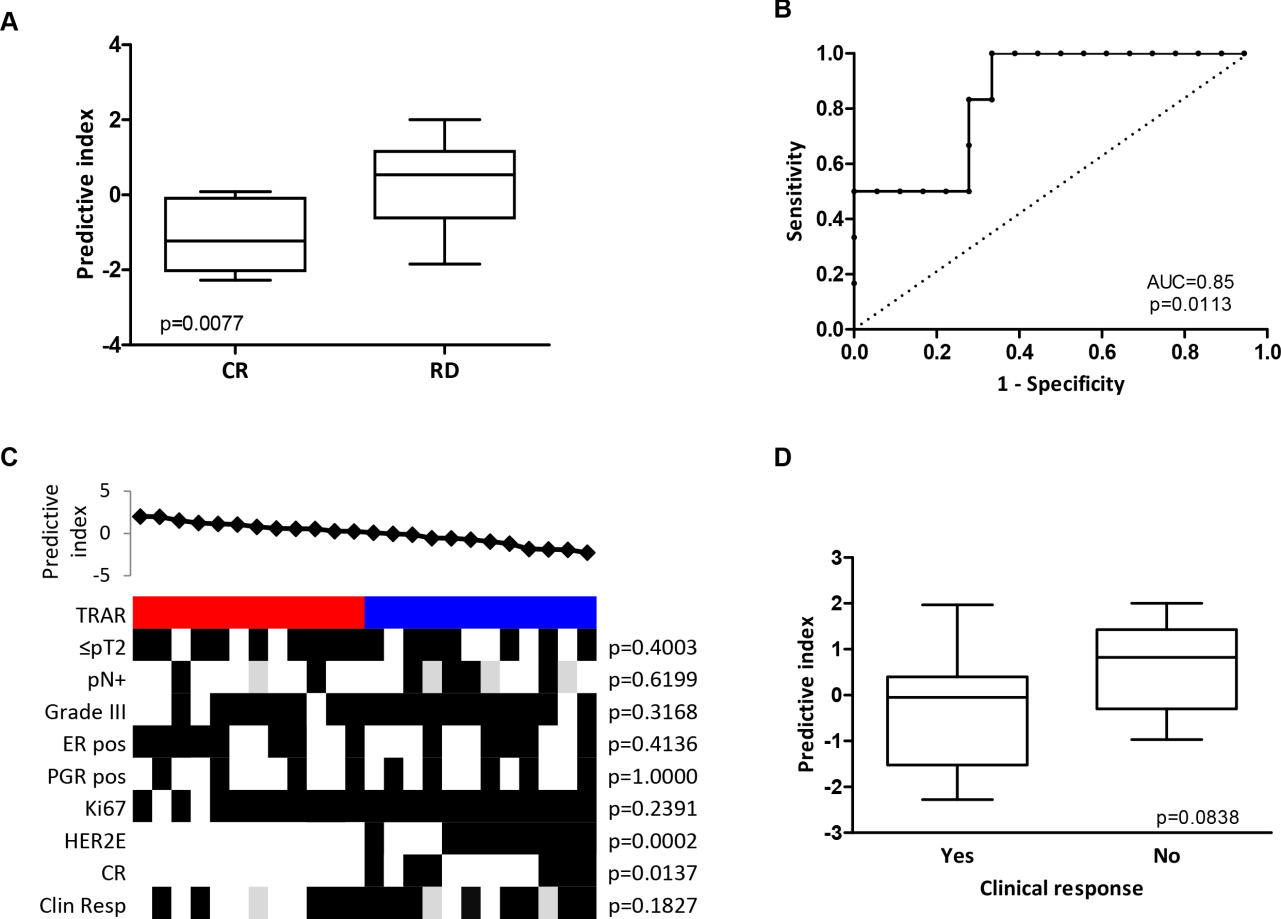
Predictive performance of TRAR model **A.** Association between TRAR predictive indices and response to trastuzumab neo-adjuvant therapy in HER2+ BCs of the TRUP cohort. CR: pathological complete response (*n* = 6), RD: residual disease (*n* = 18). *p*-values by unpaired *t*-test. **B.** ROC curve of response prediction for the 41-gene model. AUC: Area under the ROC curve. **C.** Association between predictive indices and clinical and pathological characteristics. TRAR classification (red, high risk; blue, low risk); pN, lymph node; ER, estrogen receptor; PGR, progesterone receptor; pCR: pathological complete response; Clin Resp: clinical response. Grey boxes indicate missing data. *p*-values by Fisher’s exact test. **D.** Association between TRAR predictive indices and clinical response to one cycle of trastuzumab alone. Tumors were considered responsive (Yes, *n* = 13) when clinical dimensions were smaller after treatment than before and non-responsive (No, *n* = 8) when the opposite occurred. *p*-values by unpaired *t*-test.

To test the predictive performance of the TRAR classifier in identifying BCs that respond to neo-adjuvant trastuzumab, we analyzed the gene expression profile of 24 core biopsies obtained from tumors retrieved from the TRUP window-of-opportunity trial [9] before any treatment. Application of TRAR to this dataset showed that patients achieving a complete response (pCR) to trastuzumab-based chemotherapy had significantly lower predictive indices than those with residual disease (Figure 3A). The classifier also showed good performance in identifying patients experiencing a response (AUC = 0.85, 95% CI: 0.69–1.00, *p* = 0.0133, Figure 3B) when the cut-off obtained in the GHEA cohort was applied, with all pCR found in the TRAR-low group (*p* = 0.0137, Figure 3C). Also in this cohort of patients, TRAR correlated with PAM50 classification (Figure 3C) and identified the 2 pathological complete responses with perfect accuracy among non-HER2E tumors (*p* = 0.0273).

Note that patients whose tumors responded to one cycle of trastuzumab alone (i.e., a reduction in tumor volume) showed a borderline statistical significance toward lower predictive indices (*p* = 0.0838) than those whose tumors progressed upon trastuzumab treatment (Figure 3D).

To validate the predictive performance of the classifier in identifying BCs that respond to neo-adjuvant trastuzumab, we applied TRAR to the only two available public datasets, GSE22358 and GSE50948. In this analysis, patients achieving a complete response to trastuzumab-based chemotherapy showed significantly lower predictive indices than those with residual disease (*p* = 0.0057 and *p* = 0.0126, respectively; Figure S3). In both datasets, the classifier showed good performance in identifying patients whose tumors responded (GSE22358: AUC = 0.81, 95% CI: 0.63–0.99, *p* = 0.0057; GSE50948: AUC = 0.66, 95% CI: 0.53–0.78, *p* = 0.0180; Figure S3). Again, the TRAR and PAM50 classifications were associated, with a significantly lower expression of the TRAR predictive indices in HER2E than non-HER2E tumors (Figure S3). Moreover, TRAR also showed fair ability (even if not statistically significant) in identifying responsive tumors classified as non-HER2E by PAM50 in these two datasets (GSE22358: AUC = 0.73, 95% CI: 0.34–1.00, *p* = 0.1918; GSE50948: AUC = 0.71, 95% CI: 0.49–0.93, *p* = 0.0591). In contrast, the TRAR predictive indices of HER2+ BCs that respond or do not respond to neo-adjuvant chemotherapy alone did not differ significantly in the GSE50948 and GSE41656 datasets (Figure S3). Comparison of the predictive performance of TRAR in the chemotherapy and trastuzumab arms of GSE50948 dataset showed that TRAR had a significantly higher performance in predicting response in the trastuzumab arm (*p* = 0.0363), indicating that our model exhibits a different predictive value according to the addition of trastuzumab to chemotherapy.

### Biological features of tumors according to TRAR and PAM50 classification

**Figure 4:**
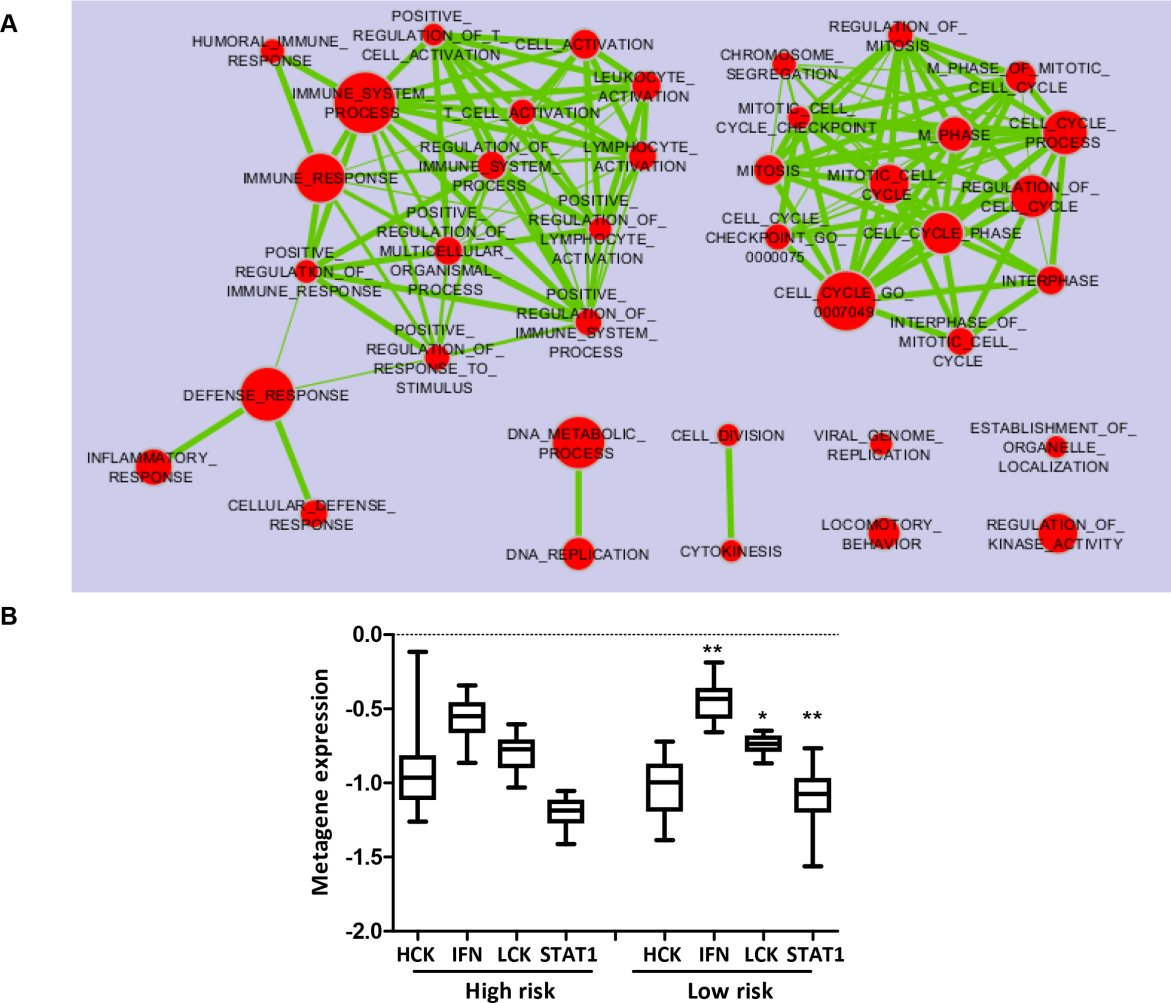
Immune metagene expression according to TRAR classification **A.** Enrichment Map of pathways (Gene Ontology Biological Processes) significantly enriched (*p* < 0.005, FDR <0.1) in TRAR-low compared to TRAR-high tumors by GSEA analysis. **B.** Association between immune metagene expression (HCK: hematopoietic cell kinase, IFN: interferon, LCK: lymphocyte-specific kinase metagenes) and TRAR subtypes. *p*-values by one-way ANOVA (*p = 0.023–0.025, **p = 0.0034).

Exploiting the whole-transcriptome profile of the GHEA cohort, we investigated the biological features of TRAR-low and -high tumors. Enrichment analysis according to the TRAR model identified several gene sets differentially and significantly enriched in the TRAR-low subgroup characterized by overexpression of immune system-related genes and proliferation-associated pathways (Figure 4A and Table S2). Further examination of immune infiltration subtypes using immune metagenes [10] in the GHEA cohort showed that compared to TRAR-high tumors, TRAR-low tumors expressed significantly higher levels of lymphocyte-specific kinase (LCK) metagene, a surrogate marker of T cells, and STAT1 and interferon (IFN) metagenes, associated with IFN signal transduction (Figure 4B). Pathological assessment of immune infiltrating cells in tumors by examining FFPE slides indicated no differences in their total amount according to TRAR classification, and immunohistochemical characterization of leukocytes and B lymphocytes revealed similar percentages of CD45+ and CD20+ cells in the two groups (Table 1). No or only very low numbers (<5 cells) of NK cells were found in tumors independent of TRAR classification (data not shown). In contrast, TRAR-low tumors showed significantly higher T cell infiltration (*p* = 0.0084) and CD8+ T cell numbers than did TRAR-high tumors (Table 1 and Figure S4).

**Table 1:** Association between immune infiltrates and TRAR classification

Variable	TRAR-high *n*/tot (%)	TRAR-low *n*/tot (%)	*p-value*^Ŧ^
CD45 positive*	11/27 (41)	14/26 (54)	0.4142
CD20 positive*	11/26 (42)	12/20 (60)	0.3726
CD3 positive*	8/25 (32)	16/22 (73)	0.0084
CD8 positive*	7/27 (26)	16/24 (67)	0.0050

* Tumors were considered positive when the percentage of positive pixels/μm^2^, as evaluated in the digitalized stained slides, was higher than the median value. See methods for details.

Ŧ *p*-value calculated by Fisher’s exact test.

## DISCUSSION

Our analyses of tumors derived from patients treated in routine clinical practice identify a new molecular classifier, TRAR, predictive of adjuvant trastuzumab benefit based on the expression levels of 41 genes associated with early relapse. Unlike previous works [4, 7], we did not base our gene selection on an empirical or biological approach, but instead applied a pre-developed semi-supervised learning method [11] to our dataset. The ability of our classifier to specifically identify tumors with low or high risk of relapse upon trastuzumab-containing therapy, but not chemotherapy alone, indicates that the molecular characteristics detected are specific in predicting benefit from HER2-targeted therapies rather than benefit from chemotherapy or due to low aggressiveness of the tumor. TRAR also showed good performance in predicting the response to trastuzumab neo-adjuvant treatment, consistent with the notion that the molecular features detected by this model are indicative of anti-tumor trastuzumab activity.

Our analyses point to the relevance of simultaneous high *ERBB2* and low *ESR1* expression in dictating trastuzumab efficacy, as demonstrated by the association of these characteristics with TRAR-low cases. Thus, among ∼20, 000 genes analyzed for expression levels, mRNA levels of *ERBB2*- and *ESR1*-related genes emerge as those crucial in mirroring tumor susceptibility to anti-HER2 therapy. In this context, the association of the TRAR-low tumor subset with the HER2E intrinsic profile further supports the notion that tumors with the highest activation of HER2 signal are those most sensitive to trastuzumab. Additionally, our analysis revealed the existence of a TRAR-low subset of tumors not strictly dependent on HER2 signals (non-HER2E) that takes advantage of trastuzumab-containing treatment, suggesting that TRAR predicts trastuzumab benefit better than does PAM50. TRAR-high tumors might instead grow through signals derived from other receptors (e.g., ER, IGFR), with consequent low benefit from anti-HER2 therapy. The observation that low expression of *ESR1* is associated with good benefit from trastuzumab is consistent with several lines of evidence suggesting that ER signaling is a mediator of trastuzumab resistance [12].

The whole-transcriptome approach also indicated that TRAR-low tumors are enriched in expression of immune-associated pathways, pointing to the existence in the same tumors of both HER2 dependency and immune infiltration and suggesting a possible direct relationship between the two features that would explain the ability of either feature to predict disease outcome in trastuzumab-treated patients. In this context, we found that tumors of patients who benefit from trastuzumab have significantly higher CD8+ T cells in their microenvironment, pointing for the first time in clinical samples to the relevance of these cells for trastuzumab activity, so far described only in preclinical models [13, 14]. These data, rather than supporting the exploration of the predictive performance of CD8+ cells in identifying patients who will benefit from trastuzumab, suggest that trastuzumab may elicit the anti-tumor activity of immune cells infiltrating TRAR-low tumors, in agreement with observations in tumors of other histotypes treated with inhibitors of their driver oncogenes [15].

From a clinical perspective, it is critical to understand whether trastuzumab alone is sufficient to activate the pre-existing immune microenvironment favorable to its activity in TRAR-low patients, or whether the addition of chemotherapy, reported to induce immune system activation [16], is necessary to generate this response. The response of TRAR-low tumors to one cycle of trastuzumab alone in the TRUP cohort suggests that the immune microenvironment of such BCs could support trastuzumab anti-tumor activity without chemotherapy addition.

Although further studies in an independent large cohort of patients treated with trastuzumab-containing therapy are needed to corroborate these findings, our results revealed that the therapeutic effects of trastuzumab are associated with both tumor dependence on HER2 signal and high T cell infiltration and numbers of CD8+ T cells. Comparison of the predictive performance of the two features alone or combined, as in TRAR model, will determine the best method to identify the tumors most sensitive to anti-HER2 drugs.

## MATERIALS AND METHODS

### Study design and patient cohorts

The 53 tumors profiled, with a median follow-up of 22 months, were obtained from BC patients treated with adjuvant chemotherapy plus trastuzumab between 2005 and 2009, deriving from our recent multicenter Italian observational study GHEA [17]. All patients received chemotherapy and trastuzumab at 3-week intervals for a median period of 1 year; hormone therapy was used in 53% of patients (Table S3). Relapsed samples (*n* = 23) were matched 1:1 (16 tumors) and 1:2 (7 tumors) with non-relapsed tumors (*n* = 30) for patient age, estrogen receptor positivity (ER), lymph node involvement and tumor size. All selected samples contained at least 70% tumor cells.

Twenty-four tumors of the trastuzumab-upfront in HER2 positive locally advanced breast cancer (TRUP) cohort were obtained from patients with locally advanced primary BC diagnosed using incisional biopsy and deriving from our recent prospective neo-adjuvant study [9]. Patients were treated with one cycle of trastuzumab alone followed by 4 cycles of chemotherapy and trastuzumab (Table S4). All procedures were in accordance with the Helsinki Declaration. Biospecimens used for research consisted of leftover material of samples collected during standard surgical and medical approaches at Fondazione IRCCS Istituto Nazionale dei Tumori of Milan and Istituti Ospitalieri di Cremona. Samples were donated by patients to the Institutional BioBanks for research purposes, and aliquots were allocated to this study after approval by the Institutional Review Board and a specific request to the Independent Ethical Committee of the institutes.

### Immunohistochemistry

HER2, Ki67 and ER positivity were re-evaluated by HercepTest, antibody clone MIB-1 and antibody clone EP1 (all from Dako, Hamburg, Germany), respectively, on histological sections consecutive to those used for gene expression profiling. HER2 positivity was defined as 3+ overexpression in more than 10% of tumor cells by immunohistochemical (IHC) testing or 2+ overexpression and HER2 amplification ratio of at least 2.2 by fluorescence *in situ* hybridization (FISH). Tumors were considered Ki67- and ER-positive if at least 14% and 10% of cells showed immunoreactivity, respectively.

### Immune cell infiltration evaluation

Pathological assessment of stromal lymphocytic infiltration was carried out as described [18].

Infiltration of total leukocytes, T, B, CD8+ T cells and NK cells was analyzed immunohistochemically on FFPE tumor sections using the Automated Immunostainer. The following antibodies were used: mouse anti-human CD45 (1:200, Dako), rabbit anti-human CD3 (1:400, Dako), mouse anti-human CD20 (1:400, Dako), mouse anti-human CD8 (1:200, Dako), and mouse anti-human CD56 (1:400, NeoMarkers, Fremont, CA), respectively. Antigen retrieval was carried out using the Target Retrieval Solution pH9 (Dako). Immunoreactions were visualized using streptavidin-biotin-peroxidase, followed by counterstaining with Carazzi hematoxylin. Stained slides were digitized by a slide scanner (ImageScope XT, Aperio), and the virtual slides were subsequently evaluated using the ‘positive pixel count’ algorithm of Aperio ImageScope. The percentage of positive stromal cells was calculated as the number of positive pixels/μm^2^. Data were divided into two groups (positive and negative) using median value as cut-off.

### Gene expression analysis

RNA was extracted from FFPE tissue slices of primary tumors of the GHEA cohort using the miRNeasy FFPE kit (Qiagen, Valencia, CA) according to the manufacturer’s protocol and from frozen incisional biopsies of the TRUP cohort using the miRNeasy MINI KIT (Qiagen). RNA quality was checked by pre-analytical screening using RT-qPCR as described [19]. Gene expression profiles were generated using Whole-Genome DASL (cDNA-mediated Annealing, Selection, Extension, and Ligation) assay and HumanHT12_v4 BeadChips (Illumina, San Diego, CA), according to protocol. The Illumina BeadArray Reader was used for scanning the arrays. Illumina BeadScan software was used for image acquisition and recovery of primary data, after which the data were quantile-normalized using BeadStudio software. The BeadChips cover more than 29, 000 annotated genes derived from RefSeq (Build 36.2, Release 38) and, after filtering, a data matrix for the GHEA cohort and the TRUP cohort were generated. The data were deposited at the Gene Expression Omnibus repository (accession numbers GSE55348 and GSE62327, respectively).

### Bioinformatics analysis

Bioinformatic analysis was performed using R [20], version 2.15, BioConductor [21], release 2.10, and BrB-ArrayTool developed by Dr. Richard Simon and the BRB-ArrayTools Development Team (v4.2.0, http://linus.nci.nih.gov/BRB-ArrayTools.html).

Gene-set enrichment analysis was performed using GSEA v2.0.13 [22] on GO biological processes. Genes represented by more than one probe were collapsed to the probe with the maximum value using the Collapse Dataset tool. Gene set permutation type was applied 1000 times and gene set enrichment was considered significant at *p* < 0.05, FDR <10%.

### Survival model development and performance

A survival model was developed in the GHEA53 training set. A disease-free survival profile was identified using a semi-standardized method involving principal component analysis [11]. Significance of each gene entered into the model was measured based on a univariate Cox proportional hazards regression of survival time versus the gene log expression level. A 10-fold cross-validation method was applied: 10% of the cases were omitted and for the remaining cases, the genes correlated with RFS at *p* < 0.001 were selected. Subsequently, principal component analysis was used to reduce the dimensionality of genes present in the model to capture most of their variability. The first two principal components, PC1 and PC2, were used to develop a prognostic model in which a prognostic trastuzumab risk (TRAR) score was calculated as: TRAR = α PC1 + β PC2, where α and β are the regression coefficients of the two principal components fitted by the Cox proportional hazards model in 10-fold cross-validation. Samples were classified as high- or low-risk by a 10-fold cross-validation approach: based on the median index values obtained in the training set comprising 90% of the cases, the remaining 10% of omitted test cases were classified. After reiteration of the entire procedure, omitting a different 10% of cases until each case was omitted once, all cases were stratified. Analysis and plotting were conducted using R package superpc (http://www-stat.stanford.edu/∼tibs/superpc).

Prediction accuracy was evaluated through time-dependent ROC curves at maximum time points of follow-up using the SurvJamda R package [23]. ROC curve assessment for censored survival data was performed using the non-parametric estimator based on nearest neighbor bivariate distribution [24]. Accuracy of the prediction was plotted after 10-fold cross-validation and reported as mean and standard deviation.

Validation of the TRAR model was carried out as described [25] on the TRUP dataset. Briefly, the training and the validation sets were normalized using the empirical Bayes (EB) method [26] to build a joint dataset. This tool is designed to reduce non-biological systematic technical biases due to the use of chips belonging to different production batches. After batch correction, the entire 41-gene model was applied to the validation set without any modification.

### External datasets and signatures

TRAR was applied to HER2-positive patients of the following public datasets (see Figure 1): Metabric (treated with adjuvant chemotherapy alone) [27]; ii) GSE22358 (treated with neo-adjuvant chemotherapy and trastuzumab) [28]; iii) GSE41656 (treated with neo-adjuvant chemotherapy alone) [29]; and iv) GSE50948 (treated with neo-adjuvant chemotherapy alone or chemotherapy and trastuzumab) [5]. Microarray data were processed starting from the authors’ raw data; if raw data or processing methods were not available, the processed data were retrieved. From the Metabric dataset, 132 cases were selected by IHC and HER2-gain obtained by CNV data through Affymetrix SNP 6.0 chip SNP6 (IHC 3+ and IHC 2+ with HER2_GAIN). Raw gene expression data profiled on HumanHT12_v3 BeadChips (Illumina) were retrieved from the EMBL-EBI repository (https://www.ebi.ac.uk/ega/datasets/) and RSN-normalized using the R-package: lumi [30]. Raw data for GSE22358 and GSE41656 profiled on Agilent and Affymetrix platforms, respectively, were retrieved from the GEO repository (http://www.ncbi.nlm.nih.gov/gds/) and processed as described [28, 29].

Immune metagenes were determined based on the method of Rody et al [10]. The 569 Affymetrix ProbeSets were first mapped on the Illumina platform and, among 7 immune metagenes, 3 were excluded (MHC-I, MHC-II and IgG metagenes) due to low gene number. Principal component analysis using the pcaMethods R/Bioconductor package [31] was used for genes contained in the remaining 4 metagenes to determine the first component able to capture most of the variation in the data.

The research-based PAM50 subtype predictor was applied to the GHEA and TRUP datasets using the publicly available algorithm as described [5] after performing median centering of the PAM50 genes. For external datasets, we used PAM50 subtype calls as previously reported.

### Statistical analysis

Association among categorical variables was tested by Fisher’s exact test. Two-sided *p* < 0.05 was considered significant. Survival functions were assessed using the Kaplan-Meier estimator, while log-rank test was used to compare survival distributions; RFS was defined as the time from start of trastuzumab treatment to the first event of local, regional or distant recurrence. Pathological complete response in the TRUP cohort was defined as no residual invasive tumor or *in situ* carcinoma in the primary tumor and in the nodes. Cox proportional hazards regression models were used for survival analysis, and hazard ratios (HR) were used to quantify the effects of explanatory variables on event hazards [32].

## SUPPLEMENTAY FIGURES AND TABLES


