# Supplementary material for: Whole-transcriptome analysis links trastuzumab sensitivity of breast tumors to both HER2 dependence and immune cell infiltration
Source: Oncotarget. 2015 Jul 1;6(29):28173–82. doi: 10.18632/oncotarget.4405 (PMC4695052; doi:10.18632/oncotarget.4405)
Supplement: Supplementary file 1 [file oncotarget-06-28173-s001.pdf]

SUPPLEMENTAY FIGURES AND TABLES

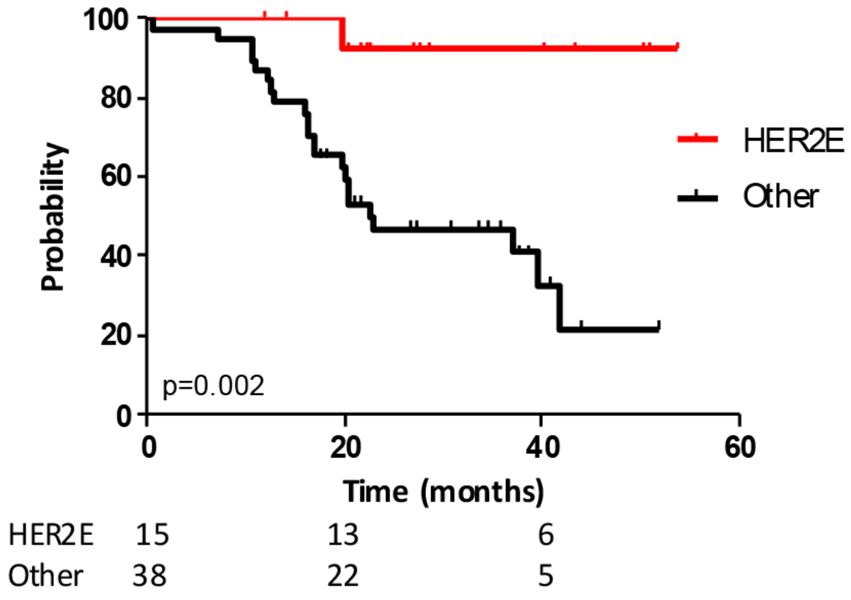

Supplementary Figure S1: Prognostic value of PAM50 subtypes in the GHEA cohort. Association between HER2E subtype (red) and other intrinsic subtypes (basal-like, luminal **A** and **B.** normal-like; black) with RFS in the GHEA53 dataset *p*-value by log-rank test.

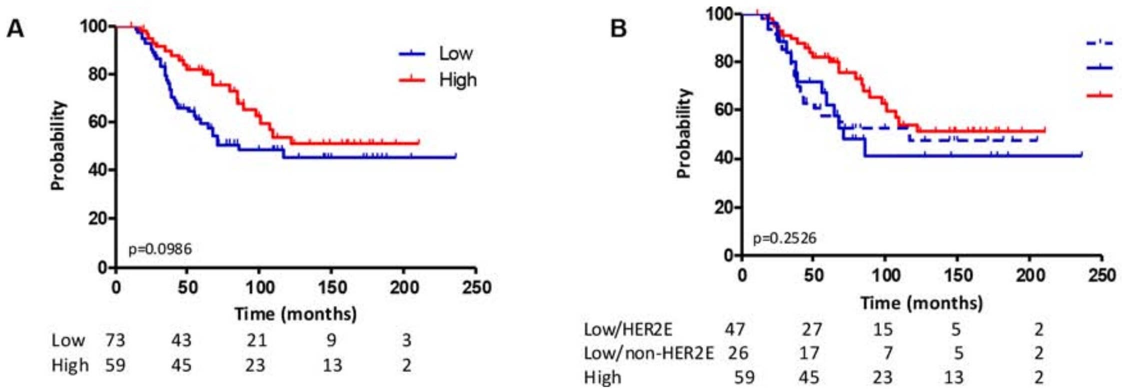

Supplementary Figure S2: Prognostic significance of TRAR model. Association between high- (red) and low-risk (blue) **A.** and high (red), low/non-HER2E (blue) and low/HER2E (dotted blue) **B.** with overall survival in HER2+ tumors of the Metabric dataset. *p*-value by log-rank test.

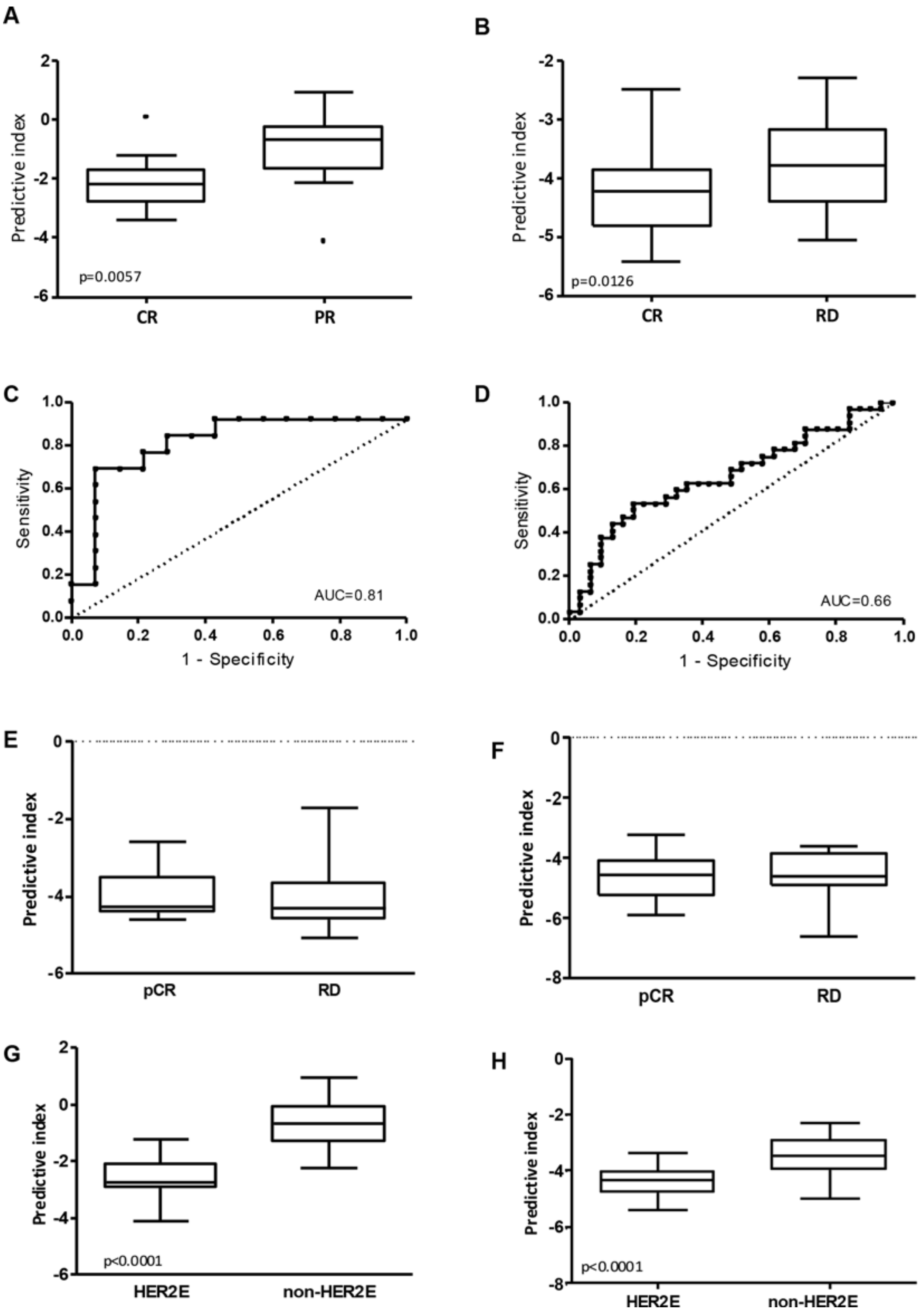

Supplementary Figure S3: Validation of TRAR predictive significance in independent datasets. A, B. Association between 41-gene predictive indices and response to trastuzumab neo-adjuvant therapy in HER2+ BCs of the GSE22358 (A) and GSE50948 (B) datasets. CR: complete or near complete response, PR: partial response RD: residual disease (number of CR GSE22358 $n = 14/27$; GSE50948 $n = 31/63$). p-values by unpaired t-test. **C, D.** Performance of the model: ROC curve of response prediction for the 41-gene model in GSE22358 (C) and GSE50948 (D) datasets. AUC: Area under the ROC curve. **E, F.** Association between TRAR indices and response to neo-adjuvant chemotherapy alone in HER2+ BCs of the GSE50948 (E) and GSE41656 (F) datasets. **G, H.** TRAR predictive indices of tumors classified as HER2E and non-HER2E in GSE22358 (G) and GSE41656 (H) datasets. p-values by unpaired t-test.

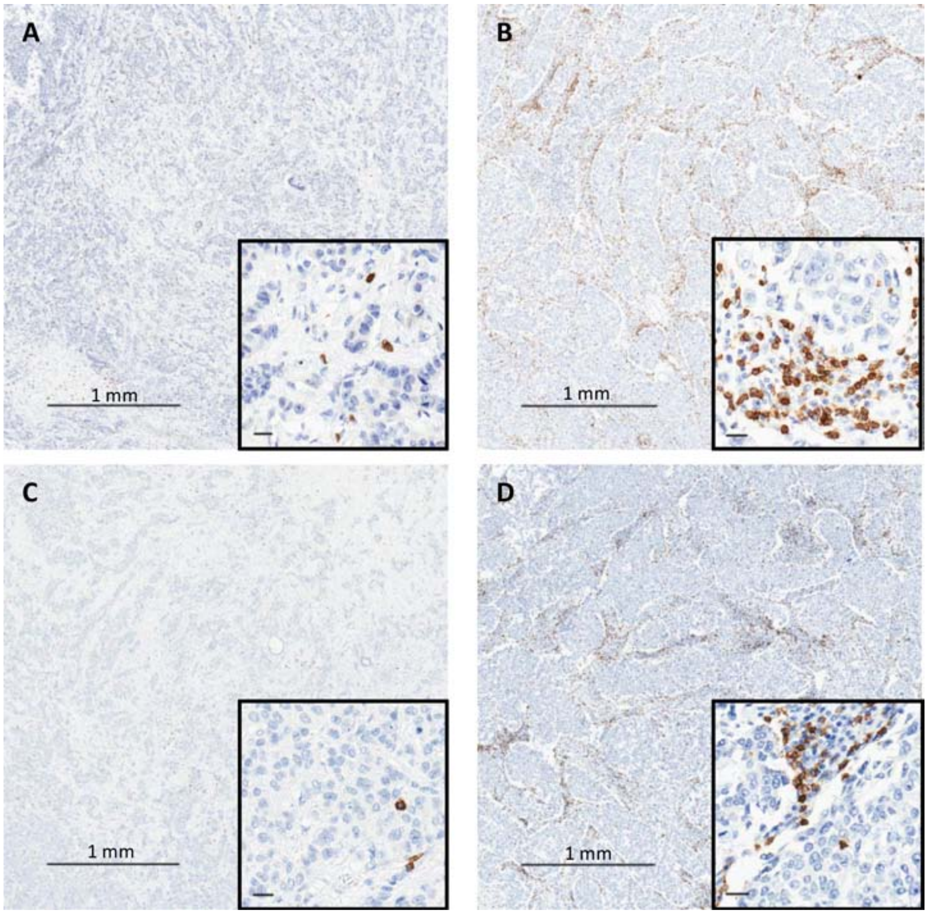

Supplementary Figure S4: Immunohistochemical evaluation of CD3- and CD8-positive cells in tissues of GHEA cohort. Representative images of CD3- and CD8-negative (**A, C.** respectively) and -positive (**B, D.** respectively) tumors. Scale bars in zoomed images, 20 µm.

Supplementary Table S1: List of 41 genes of TRAR model

Gene Symbol	Gene name
C14orf173	chromosome 14 open reading frame 173
C17orf37	chromosome 17 open reading frame 37
**C1orf186***	chromosome 1 open reading frame 186
C1QTNF7	C1q and tumor necrosis factor related protein 7
C2orf48	chromosome 2 open reading frame 48
CDK5R1	cyclin-dependent kinase 5, regulatory subunit 1 (p35)
CPA3	carboxypeptidase A3 (mast cell)
CPNE5	copine V
CTSG	cathepsin G
DBN1	drebrin 1
DERL3	derlin 3
**ERBB2***	v-erb-b2 erythroblastic leukemia viral oncogene homolog 2
**ESR1***	estrogen receptor 1
**GPC2***	glypican 2
**GRB7***	growth factor receptor-bound protein 7
HIST1H2BM	histone cluster 1, H2bm
IGFN1	immunoglobulin-like and fibronectin type III domain containing 1
INTS5	integrator complex subunit 5
KPNA5	karyopherin alpha 5 (importin alpha 6)
LRRC8D	leucine rich repeat containing 8 family, member D
MANF	mesencephalic astrocyte-derived neurotrophic factor
MEX3A	mex-3 homolog A (C. elegans)
MS4A2	membrane-spanning 4-domains, subfamily A, member 2
**ODC1***	ornithine decarboxylase 1
**ORMDL3***	ORM1-like 3 (S. cerevisiae)
PGAP3	post-GPI attachment to proteins 3
PLEKHA8	pleckstrin homology domain containing, family A member 8
PRDM1	PR domain containing 1, with ZNF domain
PSMD3	proteasome 26S subunit, non-ATPase, 3
**RERG***	RAS-like, estrogen-regulated, growth inhibitor
RGS13	regulator of G-protein signaling 13
RIPK4	receptor-interacting serine-threonine kinase 4
SLC18A2	solute carrier family 18 (vesicular monoamine), member 2
SLC8A3	solute carrier family 8 (sodium/calcium exchanger), member 3
SOX11	SRY (sex determining region Y)-box 11

(*Continued*)

Gene Symbol	Gene name
TCAP	titin-cap (telethonin)
TNFRSF17	tumor necrosis factor receptor superfamily, member 17
TPSAB1	tryptase alpha/beta 1
TPSG1	tryptase gamma 1
**VPS37B***	vacuolar protein sorting 37 homolog B (S. cerevisiae)
WASH2P	WAS protein family homolog 2 pseudogene

*TRAR core element

Supplementary Table S2: Pathways enriched in tumors according to TRAR classification by GSEA analysis

Pathway	ES	*p*-value	FDR
**TRAR-low vs TRAR-high**			
M PHASE	0.64	<0.0001	<0.0001
M PHASE OF MITOTIC CELL CYCLE	0.67	<0.0001	<0.0001
MITOSIS	0.66	<0.0001	<0.0001
IMMUNE RESPONSE	0.56	<0.0001	<0.0001
CELL CYCLE PROCESS	0.56	<0.0001	<0.0001
CELL CYCLE PHASE	0.56	<0.0001	<0.0001
IMMUNE SYSTEM PROCESS	0.52	<0.0001	<0.0001
REGULATION OF MITOSIS	0.70	<0.0001	<0.0001
MITOTIC CELL CYCLE	0.56	<0.0001	<0.0001
POSITIVE REGULATION OF IMMUNE SYSTEM PROCESS	0.67	<0.0001	0.0003
CHROMOSOME SEGREGATION	0.68	<0.0001	0.0009
POSITIVE REGULATION OF MULTICELLULAR ORGANISMAL PROCESS	0.60	<0.0001	0.0011
LYMPHOCYTE ACTIVATION	0.58	<0.0001	0.0012
CELL DIVISION	0.75	<0.0001	0.0015
REGULATION OF IMMUNE SYSTEM PROCESS	0.58	<0.0001	0.0014
CELL CYCLE GO 0007049	0.46	<0.0001	0.0017
LEUKOCYTE ACTIVATION	0.56	<0.0001	0.0035
CELL ACTIVATION	0.55	<0.0001	0.0033
DEFENSE RESPONSE	0.46	<0.0001	0.0041
T CELL ACTIVATION	0.61	<0.0001	0.0040
POSITIVE REGULATION OF IMMUNE RESPONSE	0.68	<0.0001	0.0048
POSITIVE REGULATION OF LYMPHOCYTE ACTIVATION	0.70	<0.0001	0.0046
POSITIVE REGULATION OF T CELL ACTIVATION	0.72	<0.0001	0.0049
CELLULAR DEFENSE RESPONSE	0.58	<0.0001	0.0052
INFLAMMATORY RESPONSE	0.50	<0.0001	0.0054
CYTOKINESIS	0.74	0.0038	0.0054
SISTER CHROMATID SEGREGATION	0.73	0.0058	0.0083
VIRAL GENOME REPLICATION	0.68	<0.0001	0.0098
CELL CYCLE CHECKPOINT GO 0000075	0.54	<0.0001	0.0115
ESTABLISHMENT OF ORGANELLE LOCALIZATION	0.72	0.0021	0.0121
POSITIVE REGULATION OF RESPONSE TO STIMULUS	0.56	0.0019	0.0244
LOCOMOTORY BEHAVIOR	0.48	<0.0001	0.0281
VIRAL INFECTIOUS CYCLE	0.58	0.0076	0.0287

(*continued*)

Pathway	ES	*p*-value	FDR
**TRAR-low vs TRAR-high**			
HUMORAL IMMUNE RESPONSE	0.61	0.0038	0.0322
MITOTIC CELL CYCLE CHECKPOINT	0.61	0.0019	0.0387
ORGANELLE LOCALIZATION	0.60	0.0081	0.0478
DNA RECOMBINATION	0.52	0.0059	0.0471
BIOGENIC AMINE METABOLIC PROCESS	0.66	0.0203	0.0459
CYTOKINE AND CHEMOKINE MEDIATED SIGNALING PATHWAY	0.64	0.0100	0.0505
RESPONSE TO VIRUS	0.52	0.0058	0.0508
REGULATION OF LYMPHOCYTE ACTIVATION	0.55	0.0096	0.0498
DNA REPLICATION	0.44	0.0019	0.0492
VIRAL REPRODUCTION	0.54	0.0080	0.0517
INTERPHASE OF MITOTIC CELL CYCLE	0.48	<0.0001	0.0546
REGULATION OF CELL CYCLE	0.40	<0.0001	0.0557
INTERPHASE	0.46	0.0039	0.0561
VIRAL REPRODUCTIVE PROCESS	0.55	0.0073	0.0579
RESPONSE TO OTHER ORGANISM	0.46	0.0057	0.0665
REGULATION OF T CELL ACTIVATION	0.57	0.0181	0.0666
REGULATION OF IMMUNE RESPONSE	0.55	0.0225	0.0712
DNA METABOLIC PROCESS	0.37	<0.0001	0.0731
NEGATIVE REGULATION OF DNA METABOLIC PROCESS	0.60	0.0290	0.0991
POSITIVE REGULATION OF TRANSLATION	0.54	0.0233	0.0992
REGULATION OF CYCLIN DEPENDENT PROTEIN KINASE ACTIVITY	0.48	0.0100	0.0975
REGULATION OF KINASE ACTIVITY	0.39	<0.0001	0.095
DNA INTEGRITY CHECKPOINT	0.54	0.0187	0.0947

Supplementary Table S3: Frequency of clinico-pathological characteristics of GHEA patients according to relapse

Variable	Relapsed (*n* = 23)	Non-relapsed (*n* = 30)	[Ŧ]*p*-value
Median age, y (range)	52 (32–67)	55 (35–69)	0.451
Tumor size, *T1*	8 (35%)	13 (43%)	0.581
Lymph node status, *positive*	21 (91%)	25 (83%)	0.685
Histological grade, *III*	18 (78%)	21 (70%)	0.547
Estrogen receptor, *positive*	13 (57%)	19 (63%)	0.778
Progesteron receptor, *positive*	9 (39%)	15 (50%)	0.579
Ki67, *positive*	19 (83%)	20 (67%)	0.225
Neo-adjuvant chemotherapy			
A+T+CMF	9 (39%)	7 (23%)	
CMF	0	1 (3%)	0.366
Adjuvant chemotherapy			
A	0	5 (17%)	
T	0	2 (7%)	
A+T	12 (52%)	15 (50%)	
CMF	2 (9%)	0	0.088
Hormone therapy, *yes*	12 (52%)	16 (53%)	1.000
Median trastuzumab duration, cycles (range)	18 (5–30)	18 (12–19)	0.974

[Ŧ]*p*-value is calculated by Fisher exact test.

Supplementary Table S4: Frequency of clinico-pathological characteristics of TRUP patients according to response

Variable	Responders (*n* = 6)	Non-responders (*n* = 18)	ŧ*p*-value
Tumor size, *T1 and T2*	5 (83%)	11 (61%)	0.6214
Lymph node status, *positive*	2 (50%)	6 (37%)	1.0000
Histological grade, *III*	5 (83%)	14 (78%)	1.0000
Estrogen receptor, *positive*	1 (17%)	11 (61%)	0.1550
Progesteron receptor, *positive*	1 (17%)	6 (33%)	0.6287
